# Influence of a Prostaglandin F2α Analogue on Corneal Hysteresis and Expression of Extracellular Matrix Metalloproteinases 3 and 9

**DOI:** 10.1167/tvst.12.5.28

**Published:** 2023-05-26

**Authors:** Constance Weber, Alexandra Buerger, Siegfried Priglinger, Karl Mercieca, Raffael Liegl

**Affiliations:** 1 Department of Ophthalmology, University of Bonn, Bonn, Germany; 2 Department of Ophthalmology, Ludwig-Maximilians-University, Munich, Germany

**Keywords:** prostaglandin analogues (PGA), corneal hysteresis (CH), glaucoma, extracellular matrix (ECM)

## Abstract

**Purpose:**

Low corneal hysteresis (CH) is associated with an increased risk of glaucoma. Prostaglandin analogue (PGA) eye drops may exert their intraocular pressure (IOP)-lowering effect partially by increasing CH.

**Methods:**

Twelve pairs of organ-cultured human donor corneas were used in an ex vivo model. In each case, one cornea was treated with PGA (Travoprost) for 30 days, whereas the other served as an untreated control. IOP levels were simulated in an artificial anterior chamber model. CH was measured using the Ocular Response Analyzer (ORA). Corneal expression of matrix-metalloproteinases (MMPs) was assessed by immunhistochemistry and real-time polymerase chain reaction (RT-PCR).

**Results:**

Increased CH was observed in the PGA-treated corneas. However, at IOP between 10 and 20 mm Hg, CH was increased in PGA-treated corneas (13.12 ± 0.63 mm Hg; control: 12.34 ± 0.49 mm Hg), although not significantly (*P* = 0.14). CH was significantly increased at higher IOP levels (21–40 mm Hg; PGA-treated: 17.62 ± 0.40 mm Hg; control: 11.60 ± 0.39, *P* < 0.0001). Treatment with PGA resulted in increased expression of MMP-3 and MMP-9.

**Conclusions:**

CH was increased after exposure to PGA. However, this increase was significant only in eyes with higher IOP (>21 mm Hg). A significant increase in MMP-3 and -9 was observed in PGA-treated corneas, indicating structural changes in corneal biomechanics caused by PGA.

**Translational Relevance:**

PGAs alter biomechanical structures by directly upregulating MMP-3 and -9, and the increase in CH is dependent on the level of IOP. Therefore, PGAs may have a greater effect when baseline IOP is higher.

## Introduction

Glaucoma is one of the leading causes of blindness, affecting nearly 75 million people worldwide.^1^ Elevated intraocular pressure (IOP) is one of the major risk factors for the development and progression of glaucomatous damage.^2^^,^^3^ Lowering IOP can counteract the harmful effects of glaucoma, and topical administration of pressure-lowering drugs has been shown to be an effective treatment option.^4^ Prostaglandin analogues (PGAs) are frequently used as the first-line treatment for primary open angle glaucoma (POAG) in many countries.^5^

In addition to their IOP lowering effects, PGAs have been shown to affect corneal biomechanical properties, which may also play a role in the pathophysiology of glaucoma. The Ocular Response Analyzer (ORA; Reichert Inc., Depew, NY, USA) was developed to assess clinically relevant changes in corneal biomechanics. This device measures IOP using an airstream and provides data on corneal tissue properties, including corneal hysteresis (CH).^6^ The CH value allows a description of the corneal tissue's ability to absorb and release energy during bidirectional flattening.^7^ CH has low values in all types of glaucoma and can be used as an indicator for the diagnosis and screening of glaucoma, as there is a negative correlation between IOP and CH.^8^ Lower CH values are also associated with thinner retinal nerve fiber layer (RNFL) and lower visual field indices.^9^^,^^10^

The effect of PGAs on IOP and CH has been investigated in many studies, which have shown a decrease in IOP and an increase in CH after initiation of PGA therapy.^11^^,^^12^ Studies have suggested that PGAs not only have an IOP-lowering effect, but also appear to have a direct influence on corneal properties.^13^^,^^14^ An increased expression of matrix metalloproteinases (MMPs) in eyes treated with PGAs has been demonstrated and associated with extracellular matrix (ECM) remodeling and concomitant changes in biomechanical properties of ocular tissue.^15^^,^^16^

MMP-3 and MMP-9 are two members of this enzyme group that have been shown to be particularly affected in patients with glaucoma.^17^^,^^18^

In our study, we investigated the effect of PGA on CH and examined this effect at different IOP levels using human donor corneas and the ORA in a novel ex vivo model of glaucoma. We also measured levels of MMP-3 and MMP-9 mRNA transcription in these human corneas to investigate the resulting corneal alterations and changes of the ECM.

## Materials and Methods

### Ethics

The methods used to obtain human tissue were in accordance with the Declaration of Helsinki and were approved by the local ethics committee of the University of Munich. Written informed consent was obtained from the next of kin. All donor corneas used in this study were ineligible for transplantation due to positive serology for infectious diseases or low endothelial density and would therefore have been excluded as potential grafts.

### Human Donor Corneas 

Twelve pairs of organ-cultured human donor corneas (HDCs), taken from deceased persons with no history of eye disease up to 24 hours postmortem, were obtained from the Eye Bank of Ludwig-Maximilians University of Munich. The mean age of these donor corneas was 74.3 years (range = 64.3 years −82.1 years). For each pair, one cornea was incubated in medium containing a prostaglandin F2α analogue (Travoprost 1 µg/mL) for 30 days. The medium was changed every day. The other cornea was incubated in medium containing saline without further treatment and served as an untreated control group.

### Ex Vivo Model of Hypertension, Ocular Response Analyzer, and Biomechanics

**Figure 1.**
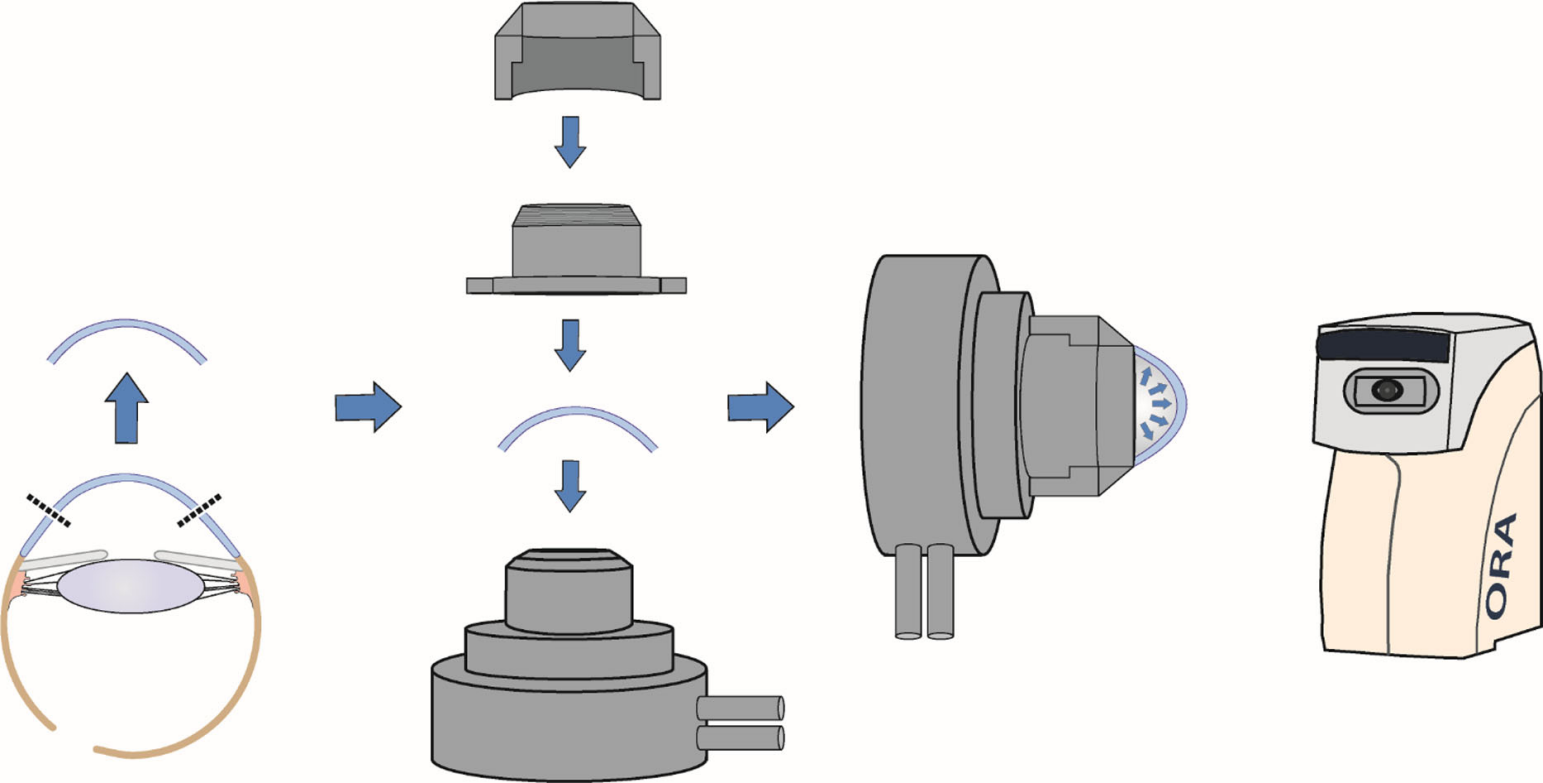
Twelve pairs of corneas were divided into 2 groups and then treated with either PGA or saline as control for 30 days. After this treatment, the corneas were placed in an artificial anterior chamber and IOP levels were set between 10 and 20 mm Hg or 21 and 40 mm Hg. Measurements were then taken using the Ocular Response Analyzer.

After 30 days of incubation, the corneas were placed in an artificial anterior chamber model (Barron Artificial Anterior Chamber; Barron Precision Instruments, Grand Blanc, MI, USA) and measurements of CH were subsequently taken. Two different ranges of IOP (10–20 or 21–40 mm Hg) were then set up. IOP values were set by the height of the water column in the infusion system and adjusted manually. This system was connected to the artificial anterior chamber model and filled with sterile saline solution. The mounted donor corneas were placed in front of the ORA machine and stable positioning was ensured. Measurements of CH were subsequently taken (Fig. 1). The setup and measurements were performed by the same team member to ensure consistency.

The ORA uses an air-pressure-triggered, dynamic, bidirectional corneal applanation process, that is detected by an electro-optical system. We obtained repeated ORA measurements and repeated every measurement three times for every cornea. CH is defined as the difference between the two intraocular pressure values measured during inward and outward applanation. Special care was taken not to damage the cornea, which could eventually result in changes of the biomechanical properties. The central corneal thickness (CCT) was measured with an ultrasound pachymetry device (Tomey SP-3000, Nagoya, Japan).

### Immunohistochemistry

After the measurements of biomechanical properties were completed, the donor corneas were fixed in 4% paraformaldehyde (Merck KGaA, Darmstadt, Germany) and 0.1% glutaraldehyde (Sigma-Aldrich Chemie GmbH, Taufkirchen, Germany) buffered in 0.1 M phosphate buffered saline (PBS) with pH 7.4 at 8°C for at least 24 hours. The corneas were then routinely processed for paraffin embedding, cut in thin (1-2 µm) sections with a sliding microtome (Reichert-Jung HN 40, Cambridge Instruments GmbH, Nussloch, Germany), and placed on Super Frost Plus microscope slides (Menzel GmbH, Braunschweig, Germany). Sections were de-paraffined with xylene (Merck), hydrated through a descending series of ethanol, and washed twice with 0.1 M PBS. In order to unmask antigens and epitopes, paraformaldehyde-fixed sections were incubated with 0.1% pepsin (Sigma-Aldrich) in 0.1 M PBS for 60 minutes and washed twice with 0.1 M PBS. To suppress nonspecific binding of IgG, specimens were incubated with normal donkey blocking serum (Dianova, Hamburg, Germany) dissolved in incubation buffer at a dilution of 1:20 for 3 hours, and washed 3 times with incubation buffer. Incubation buffer consisted of 0.1 M PBS, 0.5% bovine serum albumin (BSA; Sigma-Aldrich), 0.1% Triton-X (Sigma-Aldrich), and 0.1% sodium acid (Merck) and was used for all the following dilutions, as a wash buffer between the next steps and as a negative control. The sections were incubated overnight (18–20 hours) with primary antibodies at a dilution of 1:10, washed 3 times, and were incubated for 1 hour with fluorochrome-conjugated secondary antibodies at a dilution of 1:100 in a dark humid chamber. All chemical reactions occurred in a humid chamber at room temperature.

A rabbit polyclonal antibody against MMP-3 (sc-6839-R; Santa Cruz Biotechnology, Heidelberg, Germany) and a mouse monoclonal antibody against MMP-9 (sc-21733; Santa Cruz Biotechnology) were used as primary antibodies. The secondary antibodies were a polyclonal Cy3-conjugated donkey-anti-mouse antibody (715-165-150; Dianova) and a polyclonal Cy2-conjugated donkey-anti-rabbit antibody (711-225-152; Dianova).

After washing 4 times with incubation buffer and 3 times with 0.1 M PBS, the slides were covered with DAPI-Mounting-Medium (Dianova) and stored in darkness at 8°C. As a negative control, the respective primary antibody was omitted from the staining sequence. The histological morphology of the samples was assessed in hematoxylin and eosin (H&E)- and periodic acid Schiff (PAS)-staining sections.

### Photographic Documentation

Pictures were taken with a ProgRes CF CapturePro 2,1 optical system (Jenoptik Laser Optik Systeme GmbH, Jena, Germany) connected with the Leica DM 2500 microscope (Leica Microsystems GmbH, Wetzlar, Germany). In addition, the Leica EL6000 external light source (Leica) was used for fluorescence imaging. Pictures were taken from representative areas of the cornea especially from the chamber angle and the central and peripheral stroma, each in 10 ×, 20 ×, and 40 × magnification.

### RNA Isolation and Real-Time Polymerase Chain Reaction

Total RNA was isolated from keratocytes of human donor corneas by the guanidinium thiocyanate-phenol-chloroform extraction method (Stratagene, Heidelberg, Germany) and quantification of MMP-3 and MMP-9 mRNA was performed with specific primers using a LightCycler System (Roche Diagnostics, Mannheim, Germany) as described previously. Primers and probes were detected with ProbeFinder 2.04. The Table lists the primers used for real-time polymerase chain reaction (RT-PCR). The level of MMP-3 and MMP-9 mRNA was determined as the relative ratio (RR), which was calculated by dividing the level of the respective mRNA by the level of the 18S rRNA housekeeping gene in the same samples. The ratios are expressed as decimals. All experiments were performed in triplicate and repeated three times.

**Table.** List of Primers Used for RT-PCR.

Primers	
Name	Sequence (5′->3′)
MMP-3	F: CTGGACTCCGACACTCTGGA
	R: CAGGAAAGGTTCTGAAGTGACC
MMP-9	F: AGACCTGGGCAGATTCCAAAC
	R: CGGCAAGTCTTCCGAGTAGT

The level of MMP-3 and MMP-9 mRNA were analyzed with RT-PCR.

### Statistical Analyses

All data were analyzed with Prism 5 for Windows (Graphpad, La Jolla, CA, USA). For all statistical tests, *P* values < 0.05 were considered significant. Results of the RT-PCR were presented as mean ratios of the investigated mRNA and 18S rRNA plus the standard deviation (SD) values. All other results were presented as mean ± standard error of the mean (SEM). The Mann-Whitney *U* Test was used to analyze the results.

## Results

### ORA Measurements for Corneal Hysteresis

**Figure 2.**
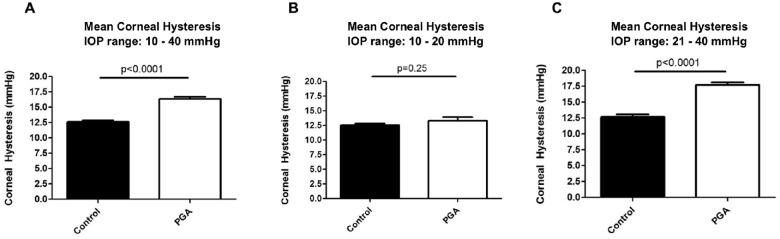
(**A**) The biomechanical measurements with ORA revealed significantly higher CH for those corneas that were treated with PGA with a mean CH of 16.62 ± 0.28 mm Hg for the PGA-treated corneas and 12.21 ± 0.27 mm Hg for the control (*P* < 0.0001). CH values differed when comparing different IOP ranges between (**B**) 10 and 20 mm Hg and (**C**) 21 and 40 mm Hg (**A**). For IOP levels of 10 to 20 mm Hg (**B**), CH was increased, but not significantly, with 13.12 ± 0.63 mm Hg for PGA treated corneas compared to 12.34 ± 0.49 mm Hg for controls (**B**: *P* = 0.14). For IOP levels of 21 to 40 mm Hg, CH was significantly higher with 17.62 ± 0.40 mm Hg for PGA-treated corneas compared to 11.60 ± 0.39 for controls (*P* < 0.0001).

Biomechanical measurements revealed a significantly higher CH in the 12 PGA-treated corneas with a value of 16.62 ± 0.28 mm Hg compared to the 12 untreated controls with 12.21 ± 1.8 mm Hg (*P* < 0.0001) for IOP levels between 10 and 40 mm Hg (Fig. 2A).

We compared CH values for the 2 different IOP levels in the anterior chamber of 10 to 20 mm Hg or 21 to 40 mm Hg for all eyes, respectively. For IOP levels between 10 and 20 mm Hg, CH was elevated in PGA-treated corneas at 13.12 ± 0.63 mm Hg in comparison to 12.34 ± 0.49 mm Hg in the control corneas (Fig. 2B). However, this difference was not significant (*P* = 0.14). For IOP levels between 21 and 40 mm Hg, CH was significantly higher in PGA-treated corneas at 17.62 ± 0.40 mm Hg compared to 11.60 ± 0.39 mm Hg (control, *P* < 0.0001; Fig. 2C).

### Pachymetry

**Figure 3.**
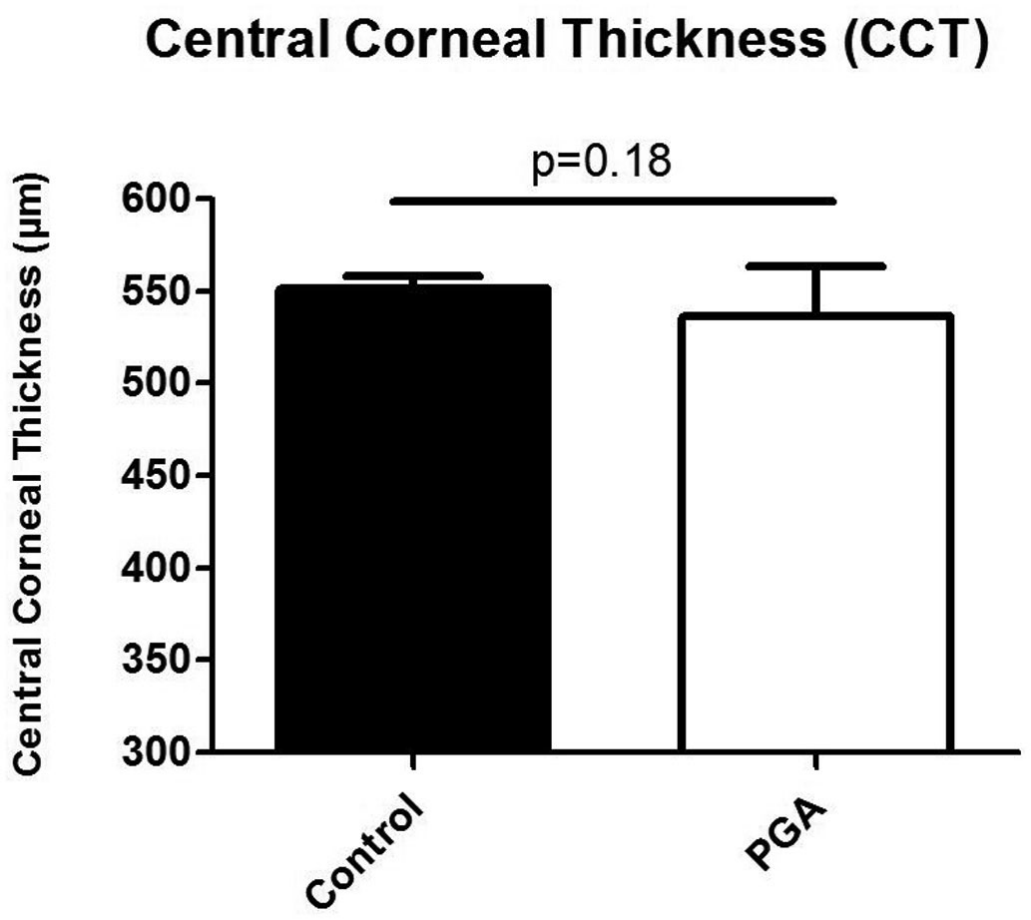
CCT was measured in all corneas with ultrasound pachymetry. We did not observe any significant differences between the two corneas when CCT was measured after 30 days. The CCT for the control group was 551.2 ± 24.49 µm and for the PGA-treated corneas 536.5 ± 27.07 µm (*P* = 0.178; *n* per group = 12).

After 30 days of incubation in our experimental setup, CCT did not differ significantly between PGA-treated corneas and untreated corneas. The CCT for the PGA-treated and untreated corneas was 536.5 ± 27.08 µm and 551.2 ± 24.49 µm, respectively (*P* = 0.1779; *n* per group = 12; Fig. 3).

### Immunohistochemistry 

**Figure 4.**
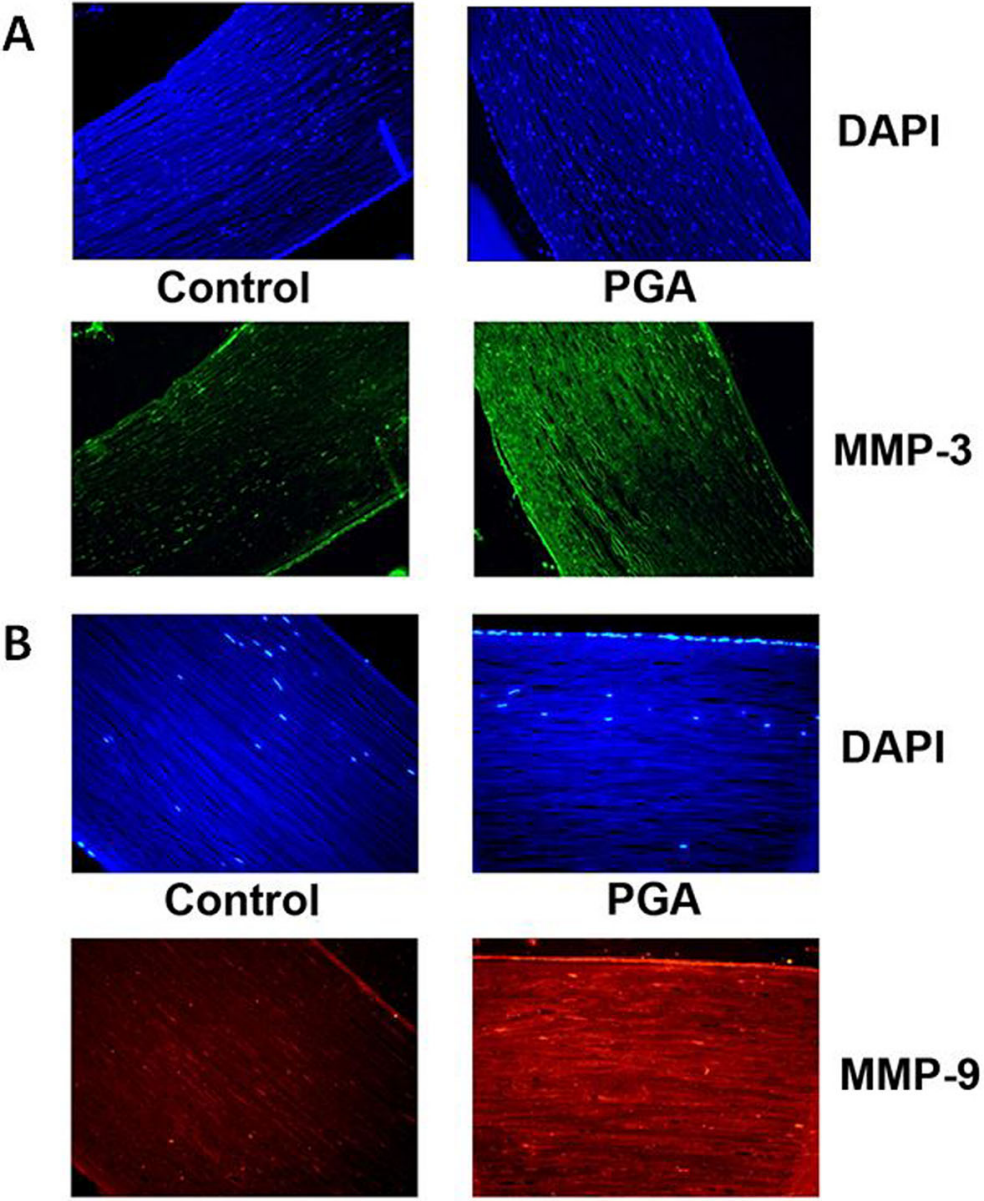
The expression of MMP-3 and MMP-9 in corneas treated with PGA for 30 days was markedly increased in immunohistochemically stained corneas. In contrast, untreated corneas, which served as controls, showed only weak staining of MMP-3 (**A**) and MMP-9 (**B**).

To detect changes in corneal biomechanics, we examined the expression of MMP-3 and MMP-9. PGA-treated corneas had an increase in MMP-3 and MMP-9 within immunohistochemically stained corneas, whereas the control corneas had a weaker staining (Fig. 4).

### Quantitative RT-PCR

**Figure 5.**
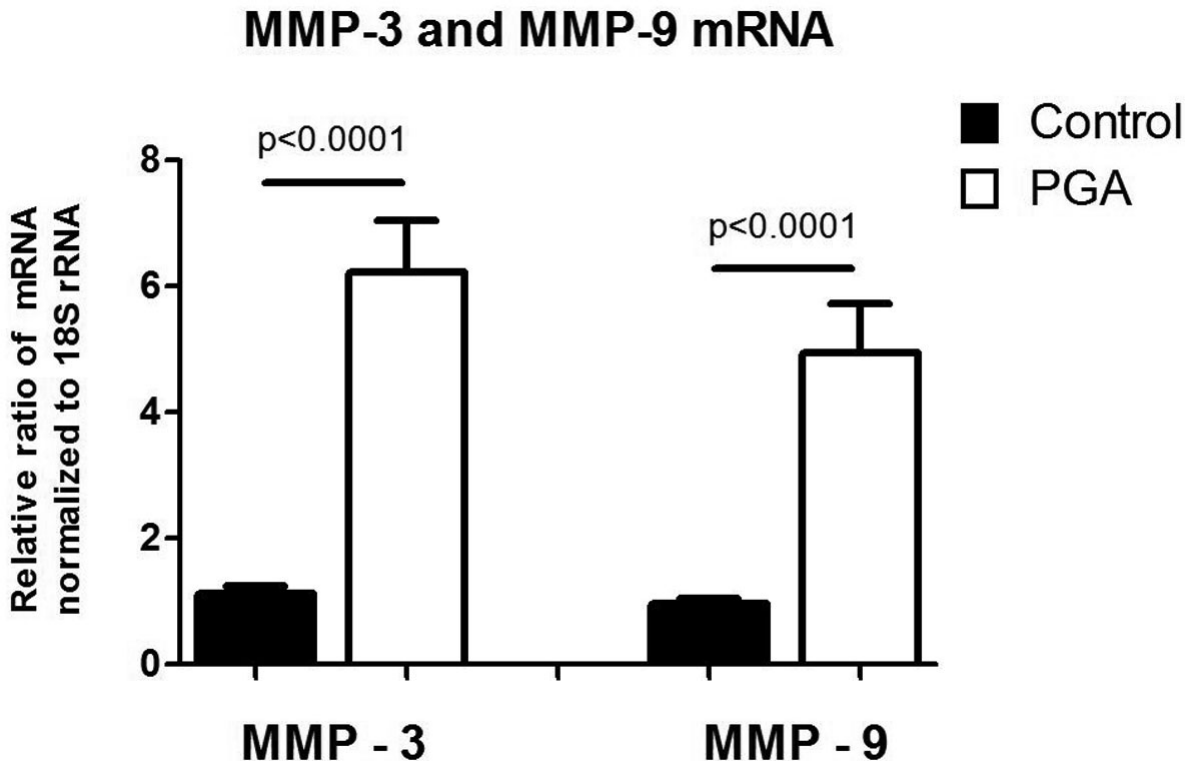
Real-time quantitative PCR was performed to measure differences in the expression of MMP-3 and MMP-9, important regulators of tissue rigidity. We observed a significant increase in the expression of MMP-3 and MMP-9 in the PGA treated corneas (*P* < 0.0001).

Significant differences in the expression of MMP-3 and MMP-9 were observed between PGA-treated corneas in comparison to the control group. A significant increase in the expression of both MMP-3 and MMP-9 was found in PGA-treated corneas (*P* < 0.0001). This increase was more than 6-fold for MMP-3, whereas it was slightly lower for MMP-9 with an increase of around 5-fold (Fig. 5).

## Discussion

To the best of our knowledge, no other study has modulated the effect of PGA in an ex vivo experiment and, in particular, examined its dependence on different IOP levels. Numerous studies have shown that PGA can decrease the IOP by up to 40% in POAG.^19^^,^^20^ Its main effect is thought to be due to an increase of uveoscleral outflow.^21^ However, several studies have shown that PGAs can also alter biomechanical properties of eye tissues and lead to alterations of corneal structures and ECM.^15^^,^^17^^,^^22^^,^^23^ CH has been postulated as an indirect measure of corneal rigidity and structure, and serves as a value that has gained increasing interest in the diagnosis and treatment of glaucoma.^24^ We demonstrated that PGA treatment significantly increased CH by approximately 25% compared to our control group (16.62 ± 0.28 mm Hg vs. 12.21 ± 1.8 mm Hg, *P* < 0.0001). Several studies have shown that PGA treatment leads to an increase in CH with a concomitant decrease in IOP. Our results confirm these findings from several in vivo studies.^11^^,^^13^^,^^25^^,^^26^

The aim of our study was to investigate the effect of PGA on CH in human corneas in an ex vivo experiment. To ensure sensitive IOP measurements, we checked for differences with respect to CCT between both groups, which showed no statistically significant differences, so that adjustments of IOP measurement to CCT were not necessary. We modulated our anterior chamber model with two different IOP ranges in order to compare the effect of PGA at different IOP levels because, to the best of our knowledge, no other study has examined its dependence on different IOP ranges. When the IOP in the artificial anterior chamber was set at 10 to 20 mm Hg, a trend toward increased CH values was found under treatment with PGA, although not significantly (13.12 ± 0.63 mm Hg vs. 12.34 ± 0.49 mm Hg, *P* = 0.14). In contrast, there was a significant difference with significant CH increase when the IOP was increased to values between 21 and 40 mm Hg (17.62 ± 0.40 mm Hg vs. 11.60 ± 0.39 mm Hg, *P* < 0.0001). Thus, we demonstrated that the effect of PGA on increasing CH was largely dependent on IOP. Therefore, it could be concluded that PGAs have a greater effect when the baseline IOP is higher.

To the best of our knowledge, this IOP-dependance of PGA is confirmed and mentioned only in a study by Aspberg and colleagues and has not been described further. They assessed 105 eyes from 86 patients receiving IOP-lowering therapy with latanoprost and found that the mean reduction from baseline at 3 months IOP was 7.9 mm Hg (28%). This IOP reduction was highly dependent on the baseline IOP levels: If the untreated baseline IOP was 1 mm Hg higher, IOP reduction increased by 0.55 mm Hg, respectively.^27^

From these results and findings of our study, it can be concluded that PGA may have a greater effect at higher IOP levels in order to effectively treat glaucoma. However, further in vivo studies are needed to elaborate further on this topic.

Studies have suggested that PGAs not only lead to a decrease in IOP, but also have a direct effect on the structural integrity of the cornea. Bolivar and colleagues conducted a prospective study of 68 eyes showing that the increase in CH was not directly correlated with the drug-induced decrease in IOP under therapy with latanoprost. They therefore hypothesized that latanoprost directly affected corneal properties.^13^ Radcliffe and colleagues compared the effect of PGA and timolol on CH showing that CH was significantly affected by PGA, whereas it remained unchanged under therapy with timolol. They attributed this difference to the redistribution of corneal stress under PGA therapy.^14^ These studies all seem to indicate a direct effect on corneal biomechanics in addition to isolated IOPs. To further investigate whether structural changes under PGA therapy were present in our ex vivo experiment, we examined the expression of MMPs as these are commonly associated with changes in corneal structures.^15^^,^^16^ MMPs degrade almost all proteins and thus play an important role in ECM turnover and thus corneal biomechanics.^28^ MMP-3 and MMP-9 are 2 members of this enzyme group, and their presence has been found to be particularly influenced by the presence of glaucoma.^17^ We demonstrated that the expression of MMP-3 and MMP-9 was significantly increased in corneas pretreated with PGA compared with untreated controls. Both qualitative immunohistochemical experiments showed that protein expression of MMP-3 and MMP-9 (see Fig. 4) was increased in PGA-treated corneas. Quantitative mRNA measurements using RT-qPCR confirmed these findings.

Weinreb and colleagues performed a review of MMPs and glaucoma treatment in 2020. They described that PGAs increase MMP expression, and their effect depends on identity and concentration.^29^ Heo and colleagues conducted a study investigating the effect of PGAs (latanoprost, bimatoprost, and unoprostone) and showed that all 3 PGAs increased MMP-1 and -9. Bimatoprost and latanoprost both increased MMP-3, whereas the effect of unoprostone was indeterminate.^30^ Ooi and colleagues reported similar results showing that the same PGAs increased MMP-1, -3, and -9.^31^

In our study, we demonstrated that PGAs lead to an increased MMP-expression in an ex vivo model. PGAs could therefore cause remodeling of the ECM, leading to an increase in CH with a decrease in IOP.

There are some limiting factors in our study. These include that the data presented were derived from an experimental approach and the number of corneas studied was relatively small. In addition, we included human donor corneas that were excluded from transplantation, because of positive serology for infectious diseases or low endothelial density. These circumstances may have influenced the PGA-induced changes in the biomechanical structures described in our study. We did not use different doses of PGA, so we were not able to investigate dose-dependent responses.

Moreover, we measured CH values only at 2 different IOP levels, ranging from 10 to 20 mm Hg and 21 to 40 mm Hg. Because of the limited number of donor corneas to which we had access, we had to limit the number of groups in order to obtain a statistically sufficient number of corneas in each group. Further, it was not possible to adjust IOP to within 1 mm Hg using the anterior chamber model. For these reasons, we chose the above IOP levels because we felt that 10 to 20 mm Hg was a good range for “normal” IOP, whereas the values above would represent high IOP, so we would end up with only 2 groups for comparison. However, these two unequal ranges would be too large to consider them for treatment comparisons in a real-world clinical setting. Further clinical studies will be needed to evaluate the IOP-dependent effect of CH at more accurate IOP levels.

In addition, we showed an increased expression of MMP-3 and MMP-9 in immunohistochemically stained corneas in the group of PGA-treated corneas. Although we believe that immunohistochemistry (IHC) does provide useful information on protein content in tissues and allows a qualitative statement to be made, it must be caveated that no quantitative measurements can be obtained and comparisons between groups are therefore limited.

Given these limitations of our study, further experimental and clinical studies evaluating the effect of PGA on CH and MMP are needed to support our experimental findings.

To the best of our knowledge, our results are the first to show that the increase in corneal hysteresis after PGA treatment was confirmed in an ex vivo experiment and that the increase in PGA-treated corneas was dependent on IOP and not vice versa. We demonstrated a direct upregulation of MMPs as an important regulator of biomechanical properties because PGA therapy presumably leads to changes in the biomechanical structure of the cornea and thus induces a remodeling of the ECM, which could lead to an increase in corneal hysteresis with a decrease in IOP.
